# Reductions in sustained prescription opioid use within the US between 2017 and 2021

**DOI:** 10.1038/s41598-024-52032-4

**Published:** 2024-01-16

**Authors:** Andrew J. Schoenfeld, Satish Munigala, Jonathan Gong, Roman J. Schoenfeld, Amanda Banaag, Christian Coles, Tracey P. Koehlmoos

**Affiliations:** 1 Department of Orthopaedic Surgery, Center for Surgery and Public Health, Brigham and Women’s Hospital, Harvard Medical School, 75 Francis Street, Boston, MA 02115 USA; 2 Department of Preventive Medicine and Biostatistics, Uniformed Services University of the Health Sciences, 4301 Jones Bridge Road, Bethesda, MD 20814 USA; 3 Department of Orthopaedic Surgery, Brigham and Women’s Hospital, Harvard Medical School, 75 Francis Street, Boston, MA 02115 USA; 4 Medfield Public Schools, 88 South Street, Medfield, MA 02052 USA

**Keywords:** Health policy, Health services

## Abstract

Over the last decade, various efforts have been made to curtail the opioid crisis. The impact of these efforts, since the onset of the COVID-19 pandemic, has not been well characterized. We sought to develop national estimates of the prevalence of sustained prescription opioid use for a time period spanning the COVID-19 pandemic (2017–2021). We used TRICARE claims data (fiscal year 2017–2021) to identify patients who were prescription opioid non-users prior to receipt of a new opioid medication. We evaluated eligible patients for subsequent sustained prescription opioid use. The prevalence of sustained prescription opioid use during 2020–2021 was compared to 2017–2019. We performed multivariable logistic regression analyses to adjust for confounding. We performed secondary analyses that accounted for interactions between the time period and age, as well as a proxy for socioeconomic status. We determined there was a 68% reduction in the odds of sustained prescription opioid use (OR 0.32; 95% CI 0.27, 0.38; *p* < 0.001) in 2020–2021 as compared to 2017–2019. Significant reductions were identified across all US census divisions and all patient age groups. In both time periods, the plurality of encounters associated with initial receipt of an opioid that culminated in sustained prescription opioid use were associated with non-specific primary diagnoses. We found significant reductions in sustained prescription opioid use in 2020–2021 as compared to 2017–2019. The persistence of prescribing behaviors that result in issue of opioids for poorly characterized conditions remains an area of concern.

## Introduction

Despite increased awareness on the part of clinicians and the lay population, as well as government directed efforts to curtail opioid prescriptions, the US remains enmeshed in an opioid crisis. In 2019, six years after the declaration from the U.S. Department of Health and Human Services regarding an opioid epidemic, nearly 10 million US residents continued to engage in prescription opioid misuse^1^. In 2017, the total economic burden of non-prescribed opioid use was estimated to exceed $1 trillion per year^2^.

In response to an increased awareness of the opioid epidemic over the last decade, several reduction efforts have been implemented including prescription drug monitoring programs, legislation around pain clinics, inappropriate prescribing behaviors, treating and reporting drug overdose, and limiting length of opioid prescriptions^3^. Such efforts, combined with the challenges of healthcare access and reliance on virtual health services that arose in conjunction with the COVID-19 pandemic, are postulated to have exerted disparate impacts on prescription opioid utilization, non-prescribed opioid use and addiction in the community. For example, Currie et al. reported that among previously opioid naïve patients, new opioid prescriptions were reduced between March and May 2020, but quickly rebounded to pre-pandemic levels thereafter^4^. At the same time, others found that opioid prescriptions were longer and more potent in the early phase of the pandemic, with monthly overdose deaths increasing in parallel^5^. Durant et al. maintained that, in Northern New England alone, the years of potential life lost due to opioid-related deaths from 2020 to 2021 was nearly 3 times higher than that due to COVID-19 (69,502 vs. 23,525)^6^. These determinations, however, may be confounded by surveillance limitations to the first few months of the pandemic, as well as geographic restrictions.

In this context, we sought to develop national estimates of the prevalence of sustained prescription opioid use in a time window spanning the onset of the COVID-19 pandemic (2017–2021) using TRICARE insurance claims data. TRICARE, the insurance product of the Department of the Defense, provides healthcare coverage to close to 9 million beneficiaries^7,8^. The covered population has been shown in previous studies to be generalizable to the US demographic aged 18–64, with broad variation in racial and ethnic composition, socioeconomic background, vocational ability and educational attainment^8–11^. Approximately 80% of the total covered population is comprised of civilians, either dependents, retirees or medically separated individuals with a disability^7–10^. TRICARE claims have been successfully used to study aspects of sustained prescription opioid use in the past^8–11^. Surveillance for prescription medication use is complete, irrespective of the environment in which they were issued, including primary and tertiary healthcare settings^8–11^. In line with previous studies^4,12,13^, we hypothesized that the prevalence of sustained prescription opioid use would be diminished in the time period of 2020–2021 when compared to 2017–2019.

## Methods

### Data source

We used TRICARE claims data (fiscal year 2017–2021) accessed from the Military Health System Data Repository (MDR). The means by which TRICARE claims are collected, compiled and accessed through the MDR have been detailed in previous investigations^8,10^. We included patients who were prescription opioid non-users (characterized using previously validated approaches^14,15^) and who received at least one class II or III opioid agonist prescription during the study time period (identified using previously defined pharmacy codes^14^; available from the authors by request). Individuals were eligible only once in the time period under study.

### Outcome measures

Opioid non-users identified as having received an opioid prescription were assessed for sustained opioid use, defined as continuous refills of class II or III opioid medications without a lapse between prescriptions of 7 days or longer for at least 6-months^14,15^. This definition of sustained prescription opioid use follows from the work of Oleisky et al.^15^, who determined that this characterization had good fidelity for disability and pain when defining chronic opioid use. Patients who met the criteria for sustained opioid use then had the primary diagnosis associated with the first opioid prescription recorded. Diagnoses were recorded according to *International Classification of Disease, Tenth Revision* (*ICD-10*) codes. Patients with a diagnosis of cancer associated with their first opioid prescription were excluded.

### Co-variates

The records of individuals identified for consideration were abstracted for the following variables: age at the time of initial opioid prescription, race, biologic sex, US census region and census division, beneficiary category, sponsor rank, environment of care (civilian vs. federal), associated branch of service and number of co-morbidities characterized using the Charlson Co-morbidity Index (CCI). Race was characterized based on individual self-report as documented in the MDR, as White, Black (e.g. African–American), Asian/Pacific Islander, Native American/Alaskan Native, Other (e.g. Other Race and Mixed Race) and Missing. Sponsor rank was stratified based on previously published investigations as Junior Enlisted, Senior Enlisted, Junior Officers and Senior Officers. In line with previous research that supports the use of sponsor rank as a proxy for socioeconomic status, we considered junior enlisted sponsor rank as indicative of lower socioeconomic status^7,8,10^.

### Statistical analysis

The primary outcome in this investigation was the prevalence of sustained prescription opioid use, defined as the number of sustained prescription opioid users divided by the number of opioid non-users issued at least one opioid prescription. The primary predictor was the time of initial opioid prescription, with the cutoff established at March 1, 2020. In this context we compared the years 2020–2021, associated with the COVID-19 pandemic to the pre-pandemic time period of 2017–2019. All other abstracted variables were considered co-variates in adjusted analyses. Descriptive statistics were calculated for all study variables using the chi-square test for bivariate comparisons. We performed multivariable logistic regression analyses to adjust for confounding from included covariates. In all adjusted analyses, based on a previously validated approach^16^, we accounted for missing race with an imputation method using reweighted estimating equations. Aligned with previous research^7^, we included US census region in adjusted analyses to account for variation in the prevalence of COVID-19 virus and the extent of local/regional government and health department restrictions. We reported all results of regression testing as odds ratios (OR) with 95% confidence intervals (CI) and *p*-values. We established statistical significance, a-priori, for variables with OR and 95% CI exclusive of 1.0 and *p* < 0.05. We performed secondary analyses that accounted for interactions between the time period and age, as well as sponsor rank, as our proxy for socioeconomic status. In these comparisons, age 55 and over and the time period 2017–2019 and Senior Officer rank and 2017–2019 were used as the referents. All statistical testing was conducted using SAS v9.4 (SAS Inst., Cary, NC) or STATA v15.1 (STATA Corp., College Station, TX). All methods were carried out in accordance with relevant guidelines and regulations. All conduct of this research and reporting follow the STROBE guidelines. All experimental protocols were approved by our institutional committee at the Uniformed Services University of the Health Sciences prior to commencement. As this was a retrospective review of previously collected de-identified claims-based data, the need for informed consent was waived by the Institutional Review Board of the Uniformed Services University of the Health Sciences.

### Ethics approval and consent to participate

All experimental protocols were approved by our institutional committee at the Uniformed Services University of the Health Sciences prior to commencement. As this was a retrospective review of previously collected de-identified claims-based data, the need for informed consent was waived by the Institutional Review Board of the Uniformed Services University of the Health Sciences.

## Results

We identified 1,478,308 individuals who were opioid non-users and issued at least one opioid prescription between 2017 and 2021. Fifty-five percent of the cohort was male with the plurality aged 18–24 (37%) and of White race (45%). The majority of the cohort was composed of civilians (40% dependents, 15% retirees and 0.1% Other) and resided in the South (Table 1). Half the individuals were of Senior Enlisted sponsor rank and 32% were in the Junior Enlisted category. There were relatively marginal differences in the sociodemographic and clinical composition of the cohort treated in 2017–2019 as compared to 2020–2021, although most findings were statistically significant given the size of our sample.

**Table 1:** Baseline sociodemographic and clinical characteristics of the study cohort.

	**All encounters**	**2017–2019**	**2020–2021**	***p*-Value**
	**N = 1,478,308**	**N = 1,279,789**	**N = 198,519**	
	**N (%)**	**N (%)**	**N (%)**	
Sex				< 0.001
Female	668,459 (45)	576,021 (45)	92,438 (47)	
Male	809,837 (55)	703,759 (55)	106,078 (53)	
Age Group				
18–24	550,400 (37)	470,046 (37)	80,354 (40)	< 0.001
25–34	334,292 (23)	290,161 (23)	44,131 (22)	< 0.001
35–44	202,128 (14)	177,047 (14)	25,081 (13)	0.513
45–54	194,209 (13)	169,870 (13)	24,339 (12)	0.599
55 +	197,279 (13)	172,665 (13)	24,614 (12)	Reference
Race				
White	664,405 (45)	576,682 (45)	87,723 (44)	Reference
Black	198,756 (13)	174,159 (14)	24,597 (12)	< 0.001
Other	36,007 (2)	31,633 (2)	4374 (2)	< 0.001
Asian/Pacific Islander	60,646 (4)	52,934 (4)	7712 (4)	< 0.001
American Indian / Alaska Native	10,444 (1)	9149 (1)	1295 (1)	0.16
Missing	508,050 (34)	435,232 (34)	72,818 (37)	< 0.001
Beneficiary category				
Dependent	597,870 (40)	514,013 (40)	83,857 (42)	< 0.001
Retiree	223,134 (15)	194,349 (15)	28,785 (15)	0.047
Active Duty	656,338 (44)	570,591 (45)	85,747 (43)	Reference
Other	961 (0.07)	832 (0.07)	129 (0.06)	0.741
Service				
Army	574,416 (39)	497,315 (39)	77,101 (39)	Reference
Air Force	383,925 (26)	332,400 (26)	51,525 (26)	0.979
Navy	321,410 (22)	280,256 (22)	41,154 (21)	< 0.001
Marines	161,321 (11)	137,979 (11)	23,342 (12)	< 0.001
Other	37,236 (3)	31,839 (2)	5397 (3)	< 0.001
Rank				
Senior Enlisted	728,818 (50)	638,366 (50)	90,452 (46)	< 0.001
Senior Officer	91,513 (6)	78,658 (6)	12,855 (7)	Reference
Junior Officer	175,327 (12)	150,559 (12)	24,768 (13)	0.575
Junior Enlisted	474,235 (32)	404,997 (32)	69,238 (35)	< 0.001
Census Region				
Midwest	103,664 (7)	89,378 (7)	14,286 (7)	< 0.001
Northeast	49,310 (3)	41,852 (3)	7458 (4)	< 0.001
South	825,309 (56)	716,312 (56)	108,997 (55)	Reference
West	388,606 (26)	335,486 (26)	53,120 (27)	< 0.001
Other	103,435 (7)	89,890 (7)	13,545 (7)	0.317
~ missing	7984 (1)	6871 (0.54)	1113 (0.56)	0.054
Comorbidities				
0	1,130,488 (76)	972,104 (76)	158,384 (80)	Reference
1	200,527 (14)	176,758 (14)	23,769 (12)	< 0.001
2	71,631 (5)	63,159 (5)	8472 (4)	< 0.001
≥ 3	75,662 (5)	67,768 (5)	7894 (4)	< 0.001
Sustained prescription opioid use	11,748 (1)	11,052 (0.87)	596 (0.30)	< 0.001

**Figure 1:**
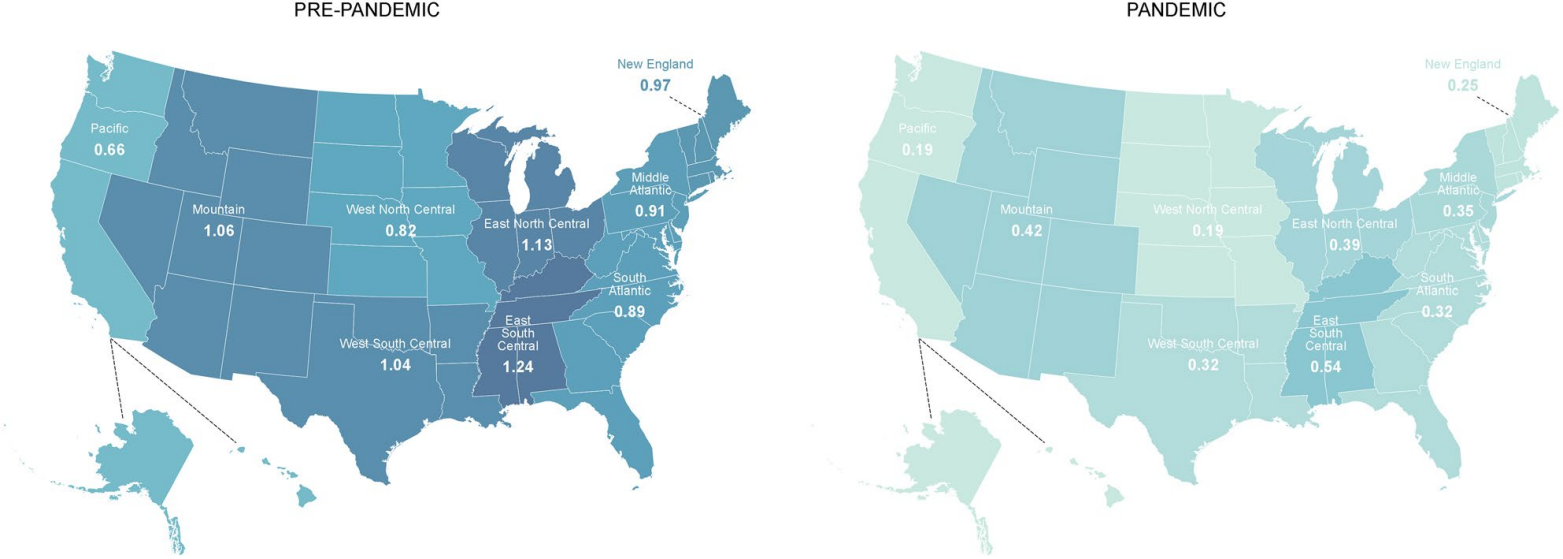
The prevalence of sustained prescription opioid use by census division in the United States from 2017 to 2019 (Pre-pandemic) compared to 2020–2021 (Pandemic).

The overall number of opioid prescriptions was significantly reduced over the course of the study period, from 49.5 per 100 individuals in the covered population in 2017–2019 to 10.4 per 100 individuals in the covered population during 2020–2021 (*p* < 0.001). Between 2017 and 2019, we found that the prevalence of sustained prescription opioid use was 0.87%. The prevalence in the time period 2020–2021 was 0.3%. In adjusted analysis accounting for all confounders, we determined there was a 68% reduction in the odds of sustained prescription opioid use (OR 0.32; 95% CI 0.27, 0.38; *p* < 0.001) in 2020–2021 as compared to 2017–2019. The prevalence of sustained prescription opioid use by census division ranged from 1.24% in the East South Central to 0.66% in the Pacific region during 2017–2019. Meaningful reductions were appreciated across all census divisions during 2020–2021 (Fig. 1), ranging from 0.54% in the East South Central to 0.19% in the Pacific region. All reductions in sustained prescription opioid use by census division, for 2020–2021 as compared to 2017–2019, were significant (*p* = 0.001 for New England; *p* < 0.001 for all other census divisions).

In both periods, the plurality of encounters associated with initial receipt of an opioid that culminated in sustained prescription opioid use were associated with non-specific primary diagnoses (R68.89, Z02.89, Z018.18, Z029, Z76.0; 39% in 2017–2019 and 44% in 2020–2021). Low back pain (3%) and obstructive sleep apnea (0.9%) were the two most common specific diagnoses in 2017–2019, while lumbar radiculopathy (1%) and chronic pain syndrome (0.8%) were the two most common in 2020–2021.

In our secondary analysis assessing for interactions between age and time period, we found significant reductions across all age groups for the period 2020–2021 as compared to individuals 55 and older in 2017–2019 as the referent (Table 2). When evaluating interactions between the time period and our proxy for socioeconomic status (Table 3), as compared to the referent, there was a significant increase in odds of sustained prescription opioid use in individuals of the lowest socioeconomic strata (e.g. Junior Enlisted; OR 1.92; 95% CI 1.51, 2.45; *p* < 0.001) during 2017–2019 (Table 3). This was reduced during 2020–2021, where no significant difference was appreciated (OR 0.75; 95% CI 0.49, 1.16; *p* = 0.19). Similarly, the odds of sustained prescription opioid use among Senior Enlisted were significantly increased during 2017–2019 (OR 2.13; 95% CI 1.75, 2.60; *p* < 0.001). However, during 2020–2021, there were significantly lower odds of sustained prescription opioid use for this subgroup (OR 0.64; 95% CI 0.48, 0.84; *p* = 0.002).

**Table 2:** Results of the multivariable logistic regression analysis regarding factors associated with the development of sustained prescription opioid use, accounting for interactions between the time period and patient age at time of receipt of initial opioid prescription.

	**Multivariable analysis**	***p*-Value**
	**Odds ratio (95% CI)**	
**Sex**		
Female	–	Reference
Male	1.21 (1.08–1.35)	0.001
Age and time period interaction		
55 + (2017–2019)	Reference	–
55 + (2020–2021)	0.35 (0.29–0.42)	< 0.001
18–24 (2017–2019)	0.16 (0.13–0.21)	< 0.001
18–24 (2020–2021)	0.12 (0.08–0.18)	< 0.001
25–34 (2017–2019)	0.64 (0.54–0.76)	< 0.001
25–34 (2020–2021)	0.28 (0.20–0.39)	< 0.001
35–44 (2017–2019)	1.10 (0.97–1.24)	0.148
35–44 (2020–2021)	0.23 (0.14–0.36)	< 0.001
45–54 (2017–2019)	0.95 (0.88–1.03)	0.187
45–54 (2020–2021)	0.31 (0.21–0.44)	< 0.001
Race		
White	–	Reference
Black	0.48 (0.44–0.54)	< 0.001
Other	0.65 (0.53–0.79)	< 0.001
Asian / Pacific Islander	0.56 (0.47–0.67)	< 0.001
American Indian / Alaska Native	0.97 (0.71–1.32)	0.856
Missing	–	–
Beneficiary category		
Active Duty	–	Reference
Dependent	2.43 (2.13–2.78)	< 0.001
Retiree	2.53 (2.27–2.87)	< 0.001
Other	4.23 (1.85–9.70)	0.001
Service		
Army	–	Reference
Airforce	0.58 (0.53–0.64)	< 0.001
Navy	0.60 (0.54–0.66)	< 0.001
Marines	0.83 (0.72–0.95)	0.009
Other	0.85 (0.68–1.06)	0.151
Rank		
Senior Officer	–	Reference
Senior Enlisted	2.13 (1.76–2.58)	< 0.001
Junior Officer	1.11 (0.87–1.39)	0.363
Junior Enlisted	1.94 (1.53–2.46)	< 0.001
Census Region		
Midwest	0.83 (0.71–0.96)	0.014
Northeast	0.79 (0.64–0.97)	0.032
South	Reference	–
West	0.94 (0.86–1.03)	0.212
Other	0.62 (0.51–0.75)	< 0.001
missing	0.34 (0.12–1.00)	0.051
Comorbidities		
None	Reference	–
1	1.77 (1.59–1.97)	< 0.001
2	2.21 (1.92–2.55)	< 0.001
≥ 3	4.02 (3.55–4.55)	< 0.001

**Table 3:** Results of the multivariable logistic regression analysis regarding factors associated with the development of sustained prescription opioid use, accounting for interactions between the time period and sponsor rank, our proxy for socioeconomic status.

	**Multivariable analysis**	***p*-Value**
	**Odds ratio (95% CI)**	
Sex		
Female	–	Reference
Male	1.21 (1.08–1.35)	0.001
Age		
55 +	Reference	–
18–24	0.17 (0.14–0.22)	< 0.001
25–34	0.65 (0.55–0.76)	< 0.001
35–44	1.08 (0.95–1.22)	0.236
45–54	0.95 (0.88–1.02)	0.152
Race		
White	–	Reference
Black	0.48 (0.44–0.54)	< 0.001
Other	0.65 (0.53–0.79)	< 0.001
Asian / Pacific Islander	0.57 (0.47–0.67)	< 0.001
American Indian / Alaska Native	0.97 (0.72–1.32)	0.859
Missing	–	–
Beneficiary Category		
Active Duty	–	Reference
Dependent	2.43 (2.13–2.78)	< 0.001
Retiree	2.53 (2.23–2.86)	< 0.001
Other	4.23 (1.85–9.70)	0.001
Service		
Army	–	Reference
Airforce	0.58 (0.53–0.64)	< 0.001
Navy	0.60 (0.54–0.66)	< 0.001
Marines	0.83 (0.72–0.95)	0.009
Other	0.85 (0.68–1.06)	0.151
Rank		
Senior Officer (2017–2019)	–	Reference
Senior Enlisted (2017–2019)	2.13 (1.75–2.60)	< 0.001
Junior Officer (2017–2019)	1.10 (0.87–1.39)	0.433
Junior Enlisted (2017–2019)	1.92 (1.51–2.45)	< 0.001
Senior Officer (2020–2021)	0.32 (0.13–0.75)	0.009
Senior Enlisted (2020–2021)	0.64 (0.48–0.84)	0.002
Junior Officer (2020–2021)	0.45 (0.27–0.76)	0.003
Junior Enlisted (2020–2021)	0.75 (0.49–1.16)	0.191
Census region		
Midwest	0.83 (0.71–0.96)	0.014
Northeast	0.79 (0.64–0.98)	0.032
South	Reference	–
West	0.94 (0.86–1.03)	0.213
Other	0.62 (0.52–0.76)	< 0.001
missing	0.34 (0.12–1.01)	0.051
Comorbidities		
None	Reference	–
1	1.77 (1.59–1.97)	< 0.001
2	2.21 (1.92–2.55)	< 0.001
≥ 3	4.02 (3.55–4.55)	< 0.001

## Discussion

Since the opioid crisis entered the popular consciousness in 2013, numerous efforts at the federal, state and local levels have been implemented to reduce the number of opioid medications circulating in the community and the prevalence of sustained prescription opioid use, non-prescribed opioid use and addiction^3–5,17–22^. While some putative success in reducing the number of opioid prescriptions was initially appreciated^3,4,12,13^, the ultimate effectiveness of these various initiatives has not been extensively studied in the context of COVID-19, especially in light of alterations in healthcare delivery that occurred due to the disruptions of the pandemic^6,7,12,20^. This investigation is among the first we are of aware of to consider the prevalence of sustained prescription opioid use in a national sample over a time frame that accounts for the effects of the COVID-19 pandemic.

Overall, we believe that our findings are encouraging as they demonstrate sizable reductions in sustained prescription opioid use in all census divisions, and for all sociodemographic and clinical subgroups considered, from 2017 to 2019 to 2020–2021. In particular, high-risk cohorts as characterized in the literature^9,12,17,19,22^, such as those of younger age and patients from lower socioeconomic strata, experienced significant reductions in the likelihood of sustained prescription opioid use during 2020–2021. Furthermore, it is also encouraging that as compared to the time period 2006–2014^14^, the total number of new sustained prescription opioid users in the Military Health System reduced substantially, from 117,118 in 2006–2014 to 11,648 during 2017–2021.

Nonetheless, the fact that the plurality of individuals in both cohorts who developed sustained prescription opioid use received their initial prescription for poorly characterized conditions, or ailments for which opioid prescriptions are not considered standard of care, is worrisome. These same prescribing behaviors were documented in the Military Health System during 2006–2014, where *Other ill-defined conditions* and *Encounter for administrative purposes* were among the most frequent diagnoses associated with the receipt of an opioid prescription in both military and civilian-run facilities^14^. This may stem from the fact that clinical burdens on providers lead them to default to simplified coding practices that do not require clear specificity around the rationale for the opioid medication issued. At the same time, the observed lack of improvement across the 15-year time frame spanning our investigation and that of the earlier study^14^, suggests a lack of efficacy regarding government mandated educational efforts in opioid stewardship and an opportunity for meaningful change going forward.

Our finding regarding the regional distribution of opioid prescribing behaviors is reasonably well aligned with prior studies on this topic, specifically with higher observed rates of opioid prescribing in the Southern US and the Midwest, as compared to New England and the Northeast^23^. The sociodemographic factors we identified as significantly associated with sustained prescription opioid use, especially during 2017–2019, are also similar to those encountered in previous works, including the influence of socioeconomic status^9,12,17,19,22^. We believe this consistency speaks to the external validity of our findings and their relevance to current health policy^24^.

This relevance is further strengthened by the national scope of our data, development from a very recent clinical cohort with characteristics that allow for generalization to the US demographic aged 64 and younger and the ability to capture the receipt of opioid prescriptions irrespective of residential location or the environment of care^8,10^. Our definition of sustained prescription opioid use also followed established best practices with respect to the use of a claims-based study source^14,15^. As a result, we maintain that our results hold meaning for clinicians, healthcare facilities and policy makers, and that they extend to the federal as well as civilian health sectors^24^.

The overarching findings suggest that the various regulatory initiatives reporting on and restricting the receipt of opioid medications may be making effective headway on reducing the number of opioid non-users who transition to sustained prescription opioid use across the country. Further qualitative research, or mixed methods work, within the Military Health System could potentially identify the practices and behaviors that have led to the reductions in sustained prescription opioid use during 2020–2021. These future findings may be scalable to other healthcare settings nationwide. At the same time, our results call for a narrower focus on the clinical rationale for an opioid prescription, and its alignment with best practices and recommendations regarding care. In this context, we suggest TRICARE and other insurance companies may elect to decline to cover opioid prescriptions that are not associated with an appropriate clinical diagnosis and indication. Additionally, prescribers who routinely issue opioids for inappropriate clinical reasons, or non-indicated clinical conditions, could be identified for additional educational initiatives, clinical practice guidance, or loss of designation as an approved provider. This may be particularly relevant to prescribers within census divisions where the prevalence of sustained prescription opioid use remains higher than the national average, such as the Mountain, East North Central, East South Central and Middle Atlantic states.

There are several limitations inherent to the work which should be recognized. Foremost, this remains a retrospective study using claims-based data, with all the inherent drawbacks associated with such a study design and data source. We do not have access to clinically granular data regarding the decision to issue an opioid prescription, the underlying rationale, or how these relate to the claims-based diagnosis codes reported to TRICARE. Discrepancies in coding practices and surveillance cannot be quantified and remain a potential source of bias that cannot be accounted for. Additionally, our study-specific definitions are predicated on the assumption that patients used opioid medications as directed by the prescribers and we are unable to evaluate non-prescription opioid use, diversion and consumption of illegally obtained prescription opioids, or heroin. This includes, our study specific definition of chronic opioid use, which was based on the work of Oleisky et al.^15^ Oleisky et al. maintained that 6-month of sustained use demonstrated good fidelity when defining chronic opioid use using claims-based data^15^. Chronic prescription opioid use has been defined differently in other research^25^ and the parameters used to establish the presence of this condition should be noted when making comparisons between studies. Viewed in this light, our estimations would be considered conservative.

Given the nature of our source population, the findings should not be generalized to patients aged 65 and older, those covered by Medicare, or those receiving treatment in VA facilities, as these represent specific societal subpopulations that may not share the characteristics of our sample. Similarly, our results are specific to a population of patients who were previously opioid non-users and cannot be extrapolated to long-term chronic users of opioid medications as a result. Several recent studies have suggested that the characteristics and clinical experience of individuals with longstanding histories of chronic opioid use have worsened in the last 5 years in terms of the inability to discontinue opioids, transition to non-prescription use and use of illegal formulations, as well as episodes of overdose and death ^9,17,18,20,22,24^.

In conclusion, this investigation is among the first we are aware of to comprehensively investigate the prevalence of sustained prescription opioid use among previously non-opioid using individuals in the time-period 2017–2021. We are encouraged by our findings which demonstrate temporal improvements as compared to historical reports and significant reductions in sustained prescription opioid use in 2020–2021 as compared to 2017–2019. The persistence of prescribing behaviors that result in issue of opioids for poorly characterized conditions, or ailments for which the issue of an opioid prescription is not considered the standard of care, remain an area for targeted improvement. This may warrant special focus in regions where the prevalence of sustained prescription opioid use remains higher than the national average, including the Mountain, East North Central, East South Central and Middle Atlantic states.

## Data Availability

The data that support the findings of this study are available from the Defense Health Agency but restrictions may apply to the availability of these data, which were used under a data sharing agreement for the current study, and so *are not publicly available*. Data are however available from the authors upon reasonable request and with permission of the Defense Health Agency. Data requests should be directed to the corresponding author: Dr. Schoenfeld.
